# Supplementary material for: GNAQ and GNA11 Genes: A Comprehensive Review on Oncogenesis, Prognosis and Therapeutic Opportunities in Uveal Melanoma
Source: Cancers (Basel). 2022 Jun 22;14(13):3066. doi: 10.3390/cancers14133066 (PMC9264989; doi:10.3390/cancers14133066)
Supplement: Supplementary file 1 [file cancers-14-03066-s001.zip › cancers-1751720-supplementary.pdf]

Table S1. UM-targeted therapies against *GNAQ* and *GNA11* in preclinical studies and their outcomes.

Drug	Target Mechanisms	UM Sample Types	Validation Methods	Anti-tumor Actions	References
AEB071 (Sotrastaurin)	PKC	UM cell lines (C918, Mel202, Mel270, Mel285, Mel290, MUM-2C, OCM-1, OCM-3, OMM-GN11, OMM1.3, UPMD1, and 92.1) and UM PDXs	AA, ATM, PA, PCR, and WB	Promotes apoptosis, and G1 cell cycle arrest Inhibits tumor growth, and NF-κB activity	(Chen et al., 2014; Wu, Li, et al., 2012)
AEB071 (Sotrastaurin) + BYL719 (Alpelisib)	PKC + PI3Kα	UM cell lines (C918, Mel202, Mel270, Mel290, OMM1, OMM1.3, and 92.1) and UM PDXs	AA, ATM, VA, and WB	Synergistic effect between Sotrastaurin + Alpelisib relative to monotherapies (Increase apoptosis, and inhibit and tumor growth)	(Musi et al., 2014)
AEB071 (Sotrastaurin) + CGM097	PKC + MDM2	UM cell lines (Mel202, Mel285, Mel290, MM28, MM33, MM66, MP38, MP41, MP42, MP65, OMM1, OMM2.5, and 92.1) and UM PDXs	AA, ATM, PA, and WB	Additive effect between Sotrastaurin + CGM097 relative to monotherapies (Increase apoptosis, inhibit proliferation, and tumor growth)	(Carita et al., 2016)
AEB071 (Sotrastaurin) + RAD001 (Everolimus)	PKC + mTOR			Synergistic effect between Sotrastaurin + Everolimus relative to monotherapies (Increase apoptosis, inhibit proliferation, and tumor growth)	

AEB071 (Sotrastaurin) + MEK162 (Binimetinib)	PKC + MEK	UM cell lines (C918, Mel202, Mel270, Mel285, Mel290, MM28, MM33, MM66, MP38, MP41, MP42, MP65, MUM-2C, OMM-GN11, OMM1, OMM1.3, OMM2.5, UPMD1, and 92.1) and UM PDXs	AA, ATM, PA, and WB	Strong synergy between Sotrastaurin + Binimetinib compared to monotherapies. (Promote apoptosis, inhibit proliferation, and tumor growth)	(Carita et al., 2016; Chen et al., 2014)
AEB071 (Sotrastaurin) + PD-0325901 (Mirdametinib)		UM cell lines (C918, Mel202, Mel270, Mel285, Mel290, MUM-2C, OMM-GN11, OMM1.3, UPMD1, and 92.1) and UM PDXs		Strong synergy between Sotrastaurin + Mirdametinib compared to monotherapies. (Promote apoptosis, and inhibit proliferation)	(Chen et al., 2014)

AHT956	PKC	UM cell lines (C918, Mel202, Mel270, Mel285, Mel290, MUM-2C, OMM-GN11, OMM1.3, UPMD1, and 92.1)	PA, and WB	Promotes G1 cell cycle arrest	(Chen et al., 2014)
AZD6244 (Selumetinib)	MEK1/2	UM cell lines (C918, M619, Mel202, Mel270, Mel285, Mel290, MUM-2B, MUM-2C, OCM-1, OCM-1A, OCM-3, OMM1.3, and 92.1) and metastatic UM patient tumor tissues	MA, PA, PCR, and WB	Promotes G1 cell cycle arrest<br>Inhibits migration, and proliferation	(Ambrosini et al., 2012; Wu, Zhu, et al., 2012)
AZD6244 (Selumetinib) + AZD8055	MEK1/2 + mTOR	UM cell lines (C918, Mel270, Mel290, OCM-1A, OCM-3, and 92.1) and UM PDXs	AA, ATM, BA, IHC, VA, and WB	Synergistic effect between Selumetinib + AZD8055 relative to monotherapies (Inhibit cell viability)<br>Apoptosis and tumor regression was preferentially induced in *BRAF* mutant UM cell lines and xenografts models	(Ho et al., 2012)
AZD6244 (Selumetinib) + Dacarbazine	MEK1/2 + Alkylating agent			Non-significant synergy between Selumetinib + Dacarbazine	

AZD6244 (Selumetinib) + Docetaxel<br>AZD6244 (Selumetinib) + AZ6197<br>AZD6244 (Selumetinib) + AZD2014 (Vistusertib)	MEK1/2 + Taxane<br>MEK1/2 + ERK<br>MEK1/2 + mTORC1/2	UM cell lines (MM28, MM66, MP38, MP41, MP46, and MP65) and UM PDXs	ATM, PD, PK, VA, and WB	Slight increase in ORR for Selumetinib + Docetaxel relative to monotherapies<br>Significant increase in ORR for both Selumetinib + AZ6197 and Selumetinib + AZD2014 relative to monotherapies	(Decaudin et al., 2018)
AZD6244 (Selumetinib) + Enzastaurin	MEK1/2 + PKC	UM cell lines (C918, M619, Mel202, Mel285, MUM-2B, MUM-2C, OCM-1, OCM-1A, OCM-3, OMM1.3, and 92.1)	AA, PA, and WB	Increase in antiproliferative effects for Selumetinib + Enzastaurin relative to monotherapies	(Wu, Zhu, et al., 2012)
AZD6244 (Selumetinib) + GDC0941	MEK1/2 + PI3K	UM cell lines (Mel202, Mel285, Mel290, MM28, MM66, MP38, MP41, MP46, MP65, OMM1, OMM2.5, and 92.1)	AA, PA, and WB	Synergistic effect between Selumetinib + GDC0941 relative to monotherapies<br>No induction of apoptosis	(Amirouchene-Angelozzi et al., 2016)

AZD6244 (Selumetinib) + MK2206	MEK1/2 + AKT	UM cell lines (C918, Mel202, Mel270, Mel290, OCM-1A, OMM1.3, and 92.1) and UM PDXs	AA, ATM, VA, and WB	Synergistic effect between Selumetinib + MK2206 relative to monotherapies (Promote autophagy, and inhibit tumor growth *in vivo*)	(Ambrosini et al., 2013)
Crizotinib	c-MET	UM cell lines (C918, Mel285, Mel290, OMM1, OMM1.3, and 92.1) and UM PDXs	ATM, IHC, LUCA, MA, PA, and WB	Inhibits migration, and proliferation<br>Non-significant tumor growth inhibition in UM xenografts<br>Prevents macrometastasis of UM cells from developing *in vivo*	(Surriga et al., 2013)
Enzastaurin	PKC	UM cell lines (C918, M619, Mel202, Mel285, MUM-2B, MUM-2C, OCM-1, OCM-1A, OCM-3, OMM1.3, and 92.1)	AA, PA, and WB	Promotes apoptosis, and G1 cell cycle arrest<br>Inhibits proliferation	(Wu, Zhu, et al., 2012)
Enzastaurin + U0126	PKC + MEK1/2	UM cell lines (C918, M619, Mel202, Mel285, MUM-2B, MUM-2C, OCM-1, OCM-1A, OCM-3, OMM1.3, and 92.1)	AA, PA, and WB	Increase in antiproliferative effects for Enzastaurin + U0126 relative to monotherapies	(Wu, Zhu, et al., 2012)

FR900359	GDI<br>ERK1/2	UM cell lines (HCmel12, Mel202, OCM-1A, OCM-3, OMM1.3, UM002B, and 92.1)	AA, BA, IF, LUCA, MA, PA, PCR, and WB	Promotes apoptosis, G1 cell cycle arrest, and melanocytic re-differentiation<br>Inhibits colony formation, migration, and proliferation	(Lapadula et al., 2019; Onken et al., 2018; Schrage et al., 2015)
GSK1120212 (Trametinib)	MEK1/2	UM cell lines (Mel202, MM28, MM66, MP38, MP41, MP46, OMM1.3, OMM1.5, UPMD1, and 92.1) and UM PDXs	AA, ATM, IHC, LUCA, PA, PCR, and WB	Reduced sensitivity of UM cell lines compared to CM cell lines<br>Promotes apoptosis<br>Induces tumor stasis	(Ma et al., 2021; Paradis et al., 2021)
GSK1120212 (Trametinib) + GSK2126458	MEK1/2 + PI3K	UM cell lines (Mel202, Mel270, Mel285, Mel290, OMM1, UPMD1, UPMD2, and 92.1)	AA, PA, and WB	Greater effect between Trametinib + GSK2126458 relative to monotherapies (Promotes apoptosis, and cell cycle arrest)	(Khalili et al., 2012)
GSK1120212 (Trametinib) + LSX196 (IDE196 = Darovasertib)	MEK1/2 + PKC	UM cell lines (Mel202, MM66, MP41, MP46, OMM1.3, UPMD1, and 92.1)	AA, LUCA, PA, PCR, and WB	Strong synergy between Trametinib + LSX196	(Ma et al., 2021)
GSK1120212 (Trametinib) +				Limited synergy between Trametinib + VS-4718 Vs	

VS-4718 (PND-1186)	MEK1/2 + FAK	UM cell lines (Mel202, MM28, MM66, MP38, MP41, MP46, OMM1.3, OMM1.5, UPMD1, and 92.1) and UM PDXs	AA, ATM, IHC, LUCA, PA, PCR, and WB	Synergistic effect between Trametinib + VS-4718 (Increase apoptosis, inhibit proliferation including cancer initiating cells, and induce tumor regression and cytotoxic effect in UM xenografts and liver metastasis models)	(Ma et al., 2021; Paradis et al., 2021)
GSK1120212 (Trametinib) + RAD001 (Everolimus)	MEK1/2 + mTOR	UM cell lines (Mel202, MM28, MM33, MM66, MP38, MP41, MP46, MP65, OMM1, OMM2.5, and 92.1)	AA, PA, PCR, and WB	Moderate synergy between Trametinib + Everolimus<br>No induction of apoptosis (except in 92.1 cell line)	(Amirouchene-Angelozzi et al., 2014)
NAV-2729	ARF6	UM cell lines (Mel202, and 92.1), UM PDXs, and UM patient tumor tissues	ATM, IA, IF, LUCA, NEA, PA, PCR, and WB	Inhibits colony formation, proliferation, and tumor growth	(Yoo et al., 2016)
PF562771	FAK	UM cell lines (Mel270, and OMM1.3)	IF, LUCA, PCR, VA, and WB	Inhibits cell viability	(Feng et al., 2019)

RAD001 (Everolimus)	mTOR	UM cell lines (Mel202, MM28, MM33, MM66, MP38, MP41, MP46, MP65, OMM1, OMM2.5, and 92.1), UM PDXs, and UM patient tumor tissues	AA, ATM, PA, PCR, and WB	Inhibits proliferation, and tumor growth	(Amirouchene-Angelozzi et al., 2014)
RAD001 (Everolimus) + GDC0941	mTOR + PI3K	UM cell lines (Mel202, Mel285, Mel290, MM28, MM66, MP38, MP41, MP46, MP65, OMM1, OMM2.5, and 92.1) and UM PDXs	AA, ATM, PA, and WB	Synergistic effect between Everolimus + GDC0941 relative to monotherapies (Increase apoptosis, and enhance anti-tumor activity *in vivo*)	(Amirouchene-Angelozzi et al., 2016)
VS-4718 (PND-1186)	FAK	UM cell lines (Mel202, Mel270, MM28, MP38, MP41, MP46, OMM1.3, OMM1.5, and 92.1) and UM PDXs	AA, ATM, IF, IHC, LUCA, PA, PCR, and WB	Promotes apoptosis<br>Inhibits colony formation, and proliferation<br>Reduces tumor size	(Feng et al., 2019; Paradis et al., 2021)

VS-4718 (PND-1186) + LSX196 (IDE196 = Darovasertib)	FAK + PKC	UM cell lines (Mel202, MM66, MP41, MP46, OMM1.3, UPMD1, and 92.1)	AA, LUCA, PA, PCR, and WB	Limited synergy between VS-4718 + LSX196	(Ma et al., 2021)
VS-4718 (PND-1186) + VS-6766	FAK + RAF/MEK	UM cell lines (MM28, MP38, MP41, MP46, OMM1.3, OMM1.5, and 92.1)	PA, LUCA, and WB	Synergistic antiproliferative effect for VS-4718 + VS-6766	(Paradis et al., 2021)
VS-6063 (Defactinib) + VS-6766	FAK + RAF/MEK	UM cell lines (MM28, MP38, MP41, MP46, OMM1.3, OMM1.5, and 92.1)	PA, LUCA, and WB	Synergistic antiproliferative effect for VS-6063 + VS-6766	(Paradis et al., 2021)
YM-254890	GDI	UM cell lines (Mel202, MM66, MP41, MP46, OMM1.3, UPMD1, and 92.1)	AA, LUCA, PA, PCR, and WB	Promotes apoptosis<br>Inhibits colony formation, and proliferation	(Ma et al., 2021)

Abbreviations: AA: apoptosis assay; ATM: animal tumor model; BA: binding assay; CM: cutaneous melanoma; GDI: guanine nucleotide dissociation inhibitor; IF: immunofluorescence; IHC: immunohistochemistry; LUCA: luciferase assay; MA: migration assay; NEA: nucleotide exchange assay; NF-κB: nuclear factor of kappa B; ORR: overall response rate; PA: proliferation assay; PCR: polymerase chain reaction; PD: pharmacodynamic assay; PDX: patient derived xenograft; PK: pharmacokinetic assay; VA: viability assay; WB: western blotting.

References

Ambrosini, G., Musi, E., Ho, A. L., de Stanchina, E., & Schwartz, G. K. (2013). Inhibition of mutant GNAQ signaling in uveal melanoma induces AMPK-dependent autophagic cell death. *Molecular Cancer Therapeutics*, *12*(5), 768-776. https://doi.org/10.1158/1535-7163.MCT-12-1020

Ambrosini, G., Pratilas, C. A., Qin, L.-X., Tadi, M., Surriga, O., Carvajal, R. D., & Schwartz, G. K. (2012). Identification of unique MEK-dependent genes in GNAQ mutant uveal melanoma involved in cell growth, tumor cell invasion, and MEK resistance. *Clinical Cancer Research: An Official Journal of the American Association for Cancer Research*, *18*(13), 3552-3561. https://doi.org/10.1158/1078-0432.CCR-11-3086

Amirouchene-Angelozzi, N., Frisch-Dit-Leitz, E., Carita, G., Dahmani, A., Raymondie, C., Liot, G., Gentien, D., Némati, F., Decaudin, D., Roman-Roman, S., & Schoumacher, M. (2016). The mTOR inhibitor Everolimus synergizes with the PI3K inhibitor GDC0941 to enhance anti-tumor efficacy in uveal melanoma. *Oncotarget*, *7*(17), 23633-23646. https://doi.org/10.18632/oncotarget.8054

Amirouchene-Angelozzi, N., Nemati, F., Gentien, D., Nicolas, A., Dumont, A., Carita, G., Camonis, J., Desjardins, L., Cassoux, N., Piperno-Neumann, S., Mariani, P., Sastre, X., Decaudin, D., & Roman-Roman, S. (2014). Establishment of novel cell lines recapitulating the genetic landscape of uveal melanoma and preclinical validation of mTOR as a therapeutic target. *Molecular Oncology*, *8*(8), 1508-1520. https://doi.org/10.1016/j.molonc.2014.06.004

Carita, G., Frisch-Dit-Leitz, E., Dahmani, A., Raymondie, C., Cassoux, N., Piperno-Neumann, S., Némati, F., Laurent, C., De Koning, L., Halilovic, E., Jeay, S., Wylie, A., Emery, C., Roman-Roman, S., Schoumacher, M., & Decaudin, D. (2016). Dual inhibition of protein kinase C and p53-MDM2 or PKC and mTORC1 are novel efficient therapeutic approaches for uveal melanoma. *Oncotarget*, *7*(23), 33542-33556. https://doi.org/10.18632/oncotarget.9552

Chen, X., Wu, Q., Tan, L., Porter, D., Jager, M. J., Emery, C., & Bastian, B. C. (2014). Combined PKC and MEK inhibition in uveal melanoma with GNAQ and GNA11 mutations. *Oncogene*, *33*(39), 4724-4734. https://doi.org/10.1038/onc.2013.418

Decaudin, D., El Botty, R., Diallo, B., Massonnet, G., Fleury, J., Naguez, A., Raymondie, C., Davies, E., Smith, A., Wilson, J., Howes, C., Smith, P. D., Cassoux, N., Piperno-Neumann, S., Roman-Roman, S., & Némati, F. (2018). Selumetinib-based therapy in uveal melanoma patient-derived xenografts. *Oncotarget*, *9*(31), 21674-21686. https://doi.org/10.18632/oncotarget.24670

Feng, X., Arang, N., Rigiracciolo, D. C., Lee, J. S., Yeerna, H., Wang, Z., Lubrano, S., Kishore, A., Pachter, J. A., König, G. M., Maggiolini, M., Kostenis, E., Schlaepfer, D. D., Tamayo, P., Chen, Q., Ruppin, E., & Gutkind, J. S. (2019). A Platform of Synthetic Lethal Gene Interaction Networks Reveals that the GNAQ Uveal Melanoma Oncogene Controls the Hippo Pathway through FAK. *Cancer Cell*, *35*(3), 457-472.e5. https://doi.org/10.1016/j.ccell.2019.01.009

Ho, A. L., Musi, E., Ambrosini, G., Nair, J. S., Deraje Vasudeva, S., de Stanchina, E., & Schwartz, G. K. (2012). Impact of combined mTOR and MEK inhibition in uveal melanoma is driven by tumor genotype. *PloS One*, *7*(7), e40439. https://doi.org/10.1371/journal.pone.0040439

Khalili, J. S., Yu, X., Wang, J., Hayes, B. C., Davies, M. A., Lizee, G., Esmaeli, B., & Woodman, S. E. (2012). Combination small molecule MEK and PI3K inhibition enhances uveal melanoma cell death in a mutant GNAQ- and GNA11-dependent manner. *Clinical Cancer Research: An Official Journal of the American Association for Cancer Research*, *18*(16), 4345-4355. https://doi.org/10.1158/1078-0432.CCR-11-3227

Lapadula, D., Farias, E., Randolph, C. E., Purwin, T. J., McGrath, D., Charpentier, T. H., Zhang, L., Wu, S., Terai, M., Sato, T., Tall, G. G., Zhou, N., Wedegaertner, P. B., Aplin, A. E., Aguirre-Ghiso, J., & Benovic, J. L. (2019). Effects of Oncogenic Gαq and Gα11 Inhibition by FR900359 in Uveal Melanoma. *Molecular Cancer Research: MCR*, *17*(4), 963-973. https://doi.org/10.1158/1541-7786.MCR-18-0574

Ma, J., Weng, L., Bastian, B. C., & Chen, X. (2021). Functional characterization of uveal melanoma oncogenes. *Oncogene*, *40*(4), 806-820. https://doi.org/10.1038/s41388-020-01569-5

Musi, E., Ambrosini, G., de Stanchina, E., & Schwartz, G. K. (2014). The phosphoinositide 3-kinase α selective inhibitor BYL719 enhances the effect of the protein kinase C inhibitor AEB071 in GNAQ/GNA11-mutant uveal melanoma cells. *Molecular Cancer Therapeutics*, *13*(5), 1044-1053. https://doi.org/10.1158/1535-7163.MCT-13-0550

Onken, M. D., Makepeace, C. M., Kaltenbronn, K. M., Kanai, S. M., Todd, T. D., Wang, S., Broekelmann, T. J., Rao, P. K., Cooper, J. A., & Blumer, K. J. (2018). Targeting nucleotide exchange to inhibit constitutively active G protein α subunits in cancer cells. *Science Signaling*, *11*(546), eaao6852. https://doi.org/10.1126/scisignal.aao6852

Paradis, J. S., Acosta, M., Saddawi-Konefka, R., Kishore, A., Gomes, F., Arang, N., Tiago, M., Coma, S., Lubrano, S., Wu, X., Ford, K., Day, C.-P., Merlino, G., Mali, P., Pachter, J. A., Sato, T., Aplin, A. E., & Gutkind, J. S. (2021). Synthetic Lethal Screens Reveal Cotargeting FAK and MEK as a Multimodal Precision Therapy for GNAQ-Driven Uveal Melanoma. *Clinical Cancer Research: An Official Journal of the American Association for Cancer Research*, *27*(11), 3190-3200. https://doi.org/10.1158/1078-0432.CCR-20-3363

Schrage, R., Schmitz, A.-L., Gaffal, E., Annala, S., Kehraus, S., Wenzel, D., Büllesbach, K. M., Bald, T., Inoue, A., Shinjo, Y., Galandrin, S., Shridhar, N., Hesse, M., Grundmann, M., Merten, N., Charpentier, T. H., Martz, M., Butcher, A. J., Slodczyk, T., … Kostenis, E. (2015). The experimental power of FR900359 to study Gq-regulated biological processes. *Nature Communications*, *6*, 10156. https://doi.org/10.1038/ncomms10156

Surriga, O., Rajasekhar, V. K., Ambrosini, G., Dogan, Y., Huang, R., & Schwartz, G. K. (2013). Crizotinib, a c-Met inhibitor, prevents metastasis in a metastatic uveal melanoma model. *Molecular Cancer Therapeutics*, *12*(12), 2817-2826. https://doi.org/10.1158/1535-7163.MCT-13-0499

Wu, X., Li, J., Zhu, M., Fletcher, J. A., & Hodi, F. S. (2012). Protein kinase C inhibitor AEB071 targets ocular melanoma harboring GNAQ mutations via effects on the PKC/Erk1/2 and PKC/NF-κB pathways. *Molecular Cancer Therapeutics*, *11*(9), 1905-1914. https://doi.org/10.1158/1535-7163.MCT-12-0121

Wu, X., Zhu, M., Fletcher, J. A., Giobbie-Hurder, A., & Hodi, F. S. (2012). The protein kinase C inhibitor enzastaurin exhibits antitumor activity against uveal melanoma. *PloS One*, *7*(1), e29622. https://doi.org/10.1371/journal.pone.0029622

Yoo, J. H., Shi, D. S., Grossmann, A. H., Sorensen, L. K., Tong, Z., Mleynek, T. M., Rogers, A., Zhu, W., Richards, J. R., Winter, J. M., Zhu, J., Dunn, C., Bajji, A., Shenderovich, M., Mueller, A. L., Woodman, S. E., Harbour, J. W., Thomas, K. R., Odelberg, S. J., … Li, D. Y. (2016). ARF6 Is an Actionable Node that Orchestrates Oncogenic GNAQ Signaling in Uveal Melanoma. *Cancer Cell*, *29*(6), 889-904. https://doi.org/10.1016/j.ccell.2016.04.015
